# High red blood cell distribution width levels could increase the risk of hemorrhagic transformation after intravenous thrombolysis in acute ischemic stroke patients

**DOI:** 10.18632/aging.203465

**Published:** 2021-08-27

**Authors:** Hongyang Fan, Xiaojie Liu, Sai Li, Peipei Liu, Yuxia Song, Haili Wang, Xiaojia Tang, Yuhan Luo, Jun Li, Yan Zhu, Yingzhu Chen

**Affiliations:** 1 The Neurology Department, The Affiliated Lianyungang Hospital of Xuzhou Medical University, Lianyungang 222002, Jiangsu, China; 2 Department of Neurology, Clinical Medical College, Yangzhou University, Yangzhou 225001, Jiangsu, China; 3 Dalian Medical University, Dalian 116000, Liaoning, China

**Keywords:** red blood cell distribution width, hemorrhagic transformation, intravenous thrombolysis, acute ischemic stroke

## Abstract

The association between the red blood cell distribution width (RDW) and hemorrhagic transformation (HT) after thrombolysis in acute ischemic stroke patients remains inconclusive. Our study aimed to assess whether high RDW levels are associated with the occurrence of HT after thrombolysis. Data were consecutively collected and retrospectively analyzed for stroke patients treated with thrombolysis between 1 January 2017 and 31 December 2019. The primary outcomes were the occurrence of HT and symptomatic HT. Among the 286 patients enrolled, 36 (12.6%) developed HT and15 (5.2%) were classified as symptomatic HT. Patients with high RDW levels were associated with a higher percentage of HT and symptomatic HT (P<0.05). The RDW levels in the HT and symptomatic HT groups were also greater compared with the no-HT group (P<0.001). Multivariable logistic regression analysis revealed that high RDW levels were independently associated with an increased risk of HT (adjusted odds ratio 2.5, 95 % CI, 1.74–3.83 P < 0.001). In conclusion, we found that high RDW levels may be an independent predictor of HT in stroke patients after thrombolysis.

## INTRODUCTION

Stroke is the leading cause of death and disability-adjusted life-years in China [1]. Acute ischemic stroke (AIS) is the third most common cause of disability and mortality after cardiovascular diseases and cancer [2]. Reperfusion involving the administration of intravenous recombinant tissue plasminogen activator (rt-PA) for intravenous recombinant and endovascular thrombectomy (EVT) is associated with better neurological outcomes for AIS patients [3]. Hemorrhagic transformation (HT) is defined as bleeding into an area of ischemic brain after stroke, and appears in 10% to 40% of acute ischemic stroke cases [4, 5], HT contributes to increased disability and mortality risk [6], and is believed to be either a part of the natural course of AIS or a common complication of intravenous thrombolytic therapy [7, 8]. Intravenous rt-PA therapy can induce or increase the risk for hemorrhagic transformation of ischemic lesions [9]. HT limits the use of intravenous thrombolysis (IVT), increases the risk of functional dependence and consequently decreases the benefit-risk ratio of IVT treatment.

The red blood cell distribution width (RDW) is a parameter that variability and heterogeneity in reflects red blood cell size [10]. RDW has been recognized as a potential independent risk factor for ischemic cerebrovascular disease. Several studies found that RDW can predict the occurrence, early mortality, 1-year survival, severity and functional outcomes of AIS patients [11–13]. A recent study reported that increased red cell distribution width is associated with an increased risk of HT in AIS patients [14]. However, previous reports have not thoroughly investigated the impact of RDW levels on hemorrhagic transformation in AIS patients who have undergone intravenous thrombolysis. Therefore, this study aimed to assess whether there was a relationship between RDW levels on admission and the development of HT after IVT, and to explore whether RDW on admission could be a biomarker of HT after IVT.

## RESULTS

**Figure 1:**
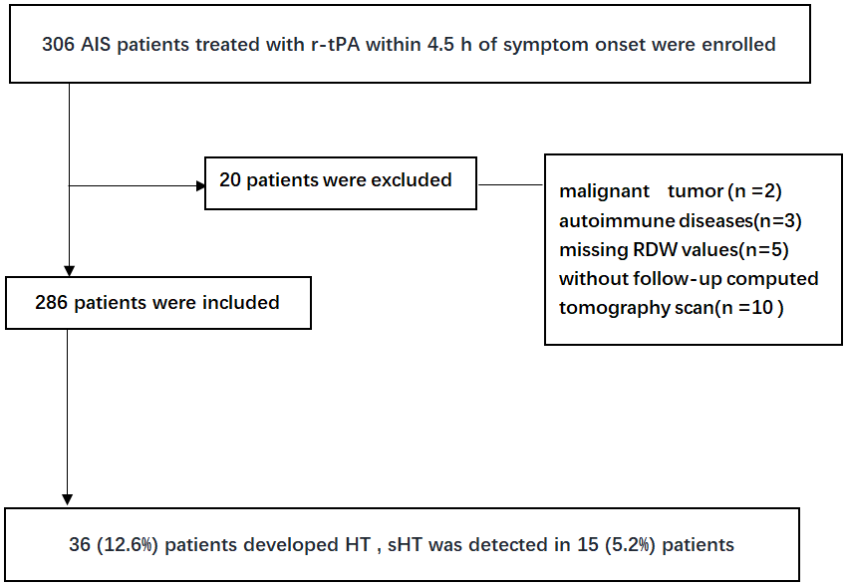
Flowchart of the study.

A total of 306 AIS patients treated with rt-PA within 4.5 h of symptom onset from Northern Jiangsu People's Hospital between 1 January 2017 and 31 December 2019 were selected to participate in this study. 20 patients were later excluded because of following reasons: malignant tumor (n =2), autoimmune diseases (n=3), lack of follow-up computed tomography scan (n=10), and 5 patients without RDW values. Consequently, data from 286 patients were eligible for the final analysis. The flowchart of the study is shown in Figure 1.

The mean age of the enrolled patients was 67.53±11.18 years (n=286). There were 171 (59.8%) men and 115 (40.2%) women. A total of 36 (12.6%) patients developed HT, and 15 (5.2%) of HT patients were classified as symptomatic HT according to the Heidelberg Bleeding Classification. The RDW levels ranged from 11.3% to 20.7% with a median of 13.1%.

Among the male patients, high RDW levels were associated with advanced age, longer onset-thrombolysis time, increased incidence of HT and symptomatic HT and low percentage of functional independence (all P< 0.05). In female patients, high RDW levels were associated with advanced age, high NIHSS score, longer onset-thrombolysis time, increased incidence of HT, and lower percentage of functional independence (all P<0.05). Characteristics of patients were shown in Table 1.

**Table 1:** The baseline demographic and clinical characteristics of the patients according to RDW level based on gender.

**Characteristics**	**Male (n= 171)**				**Female (n=115)**		
	**Tertile1**	**Tertile2**	**P-value**		**Tertile1**	**Tertile2**	**P-value**
No. (%)	86(50.29%)	85(49.71%)			58(50.43%)	57(49.57%)	
Age(years)	65.15±10.86	68.41±10.25	0.043		66.71±11.81	70.65±11.7	0.118
Hypertension(n,%)	61(70.9%)	63974.1%)	0.732		35(60.3%)	44(77.2%)	0.07
Diabetes mellitus(n, %)	23(26.7%)	15(17.6%)	0.198		13(22.4%)	14(24.6%)	0.829
Hyperlipidemia(n, %)	46(54.8%)	29(34.1%)	0.009		25(43.1%)	17(29.8%)	0.176
Consumption of alcohol	37(43%)	32(37.6%)	0.534		2(3.4%)	0(0%)	0.496
Smokers (n, %)	51(59.3%)	41(48.2%)	0.169		4(6.9%)	2(3.5%)	0.679
Previous stroke or TIA (n, %)	17(19.8%)	11(12.9%)	0.302		9(15.5%)	6(10.5%)	0.581
Atrial fibrillation(n, %)	11(12.8%)	21(24.7%)	0.052		13(22.4%)	16(28.1%)	0.525
Coronary artery disease(n, %)	7(8.1%)	8(9.4%)	0.794		5(8.6%)	9(15.8%)	0.268
Onset-thrombolysis time(minutes)	173.5 (134-210)	190(150.5-242)	0.048		207 (153.75-240)	180(130-225)	0.029
NIHSS on admission	5.99±5.78	6.22±6.05	0.607		5.41±5.8	8.95±8.98	0.019
Platelet counts(x10^9/L)	186 (157.75-222)	174(151.5-212.5)	0.224		187 (154.75-232.75)	178(136-214)	0.123
BG on admission(mmol/L)	7.16±2.98	6.34±2.26	0.077		6.69±2.42	6.48±1.9	0.963
SBP on admission (mm/Hg)	151.3±21.72	149.96±19.28	0.593		158.26±25.18	155.16±21.55	0.497
DBP on admission(mm/Hg)	87.19±14.75	86.24±12.6	0.734		85.76±13.34	85.49±13.62	0.906
HT(n, %)	4(2.9%)	32(21.9%)	<0.001		3(5.2%)	13(22.8%)	0.007
symptomatic HT(n, %)	2(1.4%)	13(8.9%)	0.005		1(1.7%)	4(7%)	0.206
Functional independence (FI)	110(78.6%)	85 (58.2%)	<0.001		43(74.1%)	28(49.1%)	0.007

TIA, Transient ischemic attack; NHISS, National Institutes of Health Stroke Scale; HT, hemorrhagic transformation; BG, blood glucose; SBP, systolic blood pressure; DBP, diastolic blood pressure; RDW, Red blood cell distribution width.

Patients with asymptomatic hemorrhagic transformation were associated with advanced age (P=0.036), and a higher frequency of atrial fibrillation (P =0.02) compared to patients with symptomatic HT. Besides, patients with symptomatic HT were associated with high NIHSS on admission (P<0.001), high RDW levels (P<0.001), low platelet counts (P=0.033) and low proportion of functional independence (P =0.002) compared to patients with asymptomatic HT and patients with no HT. As shown in Table 2.

**Table 2:** The baseline demographic and clinical characteristics of the patients according to no HT asymptomatic HT or symptomatic HT.

**Characteristics**	**No HT (250)**	**Asymptomatic HT (21)**	**Symptomatic HT (15)**	**P-value**
Age(years)	66.89±11.25	73.05±9.95	70.53±9.49	0.036
Men(n, %)	151(60.4%)	10(47.6%)	10(66.7%)	0.459
Hypertension(n,%)	176(70.4%)	14(66.7%)	13(86.7%)	0.399
Diabetes mellitus(n, %)	55(22%)	7(33.3%)	3(20%)	0.468
Hyperlipidemia(n, %)	108(43.5%)	4(19%)	5(33.3%)	0.073
Consumption of alcohol	65(26%)	1(4.8%)	5(33.3%)	0.059
Smokers(n,%)	89(35.6%)	5(23.8%)	4(26.7%)	0.452
Previous stroke or TIA (n, %)	38(15.2%)	2(9.5%)	3(20%)	0.693
Atrial fibrillation(n, %)	47(18.8%)	9(42.9%)	5(33.3%)	0.02
Coronary artery disease(n, %)	23(9.2%)	5(23.8%)	1(6.7%)	0.111
Onset-thrombolysis time(minutes)	182.5(140-225)	170(131.5-220)	233(145-250)	0.33
NIHSS on admission	4(2-7)	9(3-14.5)	12(7-23)	<0.001
RDW	13.06±0.92%	14.28±0.73%	14.55±2.04%	<0.001
Platelet counts(x10^9/L)	184(152-225)	183(151-222)	170(159-205)	0.033
BG on admission(mmol/L)	6.68±2.48	6.81±3.05	6.55±1.49	0.682
SBP on admission (mm/Hg)	154.13±22.13	143.14±18.4	149.53±19.11	0.053
DBP on admission(mm/Hg)	86.33±13.35	84.14±15.27	88.4±15.28	0.504
Functional independence (FI)	178(71.2%)	13(61.9%)	4(26.7%)	0.002

TIA, Transient ischemic attack; NHISS, National Institutes of Health Stroke Scale; HT, hemorrhagic transformation; BG, blood glucose; SBP, systolic blood pressure; DBP, diastolic blood pressure; RDW, Red blood cell distribution width.

Table 3 shows the results of univariable logistic regression analyses that were used to determine the association between all the variables and HT or sHT. The results show that age, hyperlipidemia, AF, baseline NIHSS score, admission RDW levels, admission platelet count, and SBP were significantly associated with HT. When multivariable adjustment for potential confounders was carried out, the results showed that only the RDW levels and NHISS on admission were positively associated with hemorrhagic transformation (adjusted OR 2.5, 95% CI (1.73-3.71, P < 0.001: adjusted OR:1.08 95 % CI: 1.03-1.14; P = 0.002, P <0.001; respectively). Every single point increase in NHISS increased the risk of hemorrhage transformation by 8% in AIS patients treated with intravenous thrombolysis. An increase in RDW levels by 1% increased the risk of hemorrhage transformation by 1.5. Results from univariable regression analyses in this study showed that the effect of baseline NIHSS score, admission RDW level, platelet counts on the sHT in AIS patients treated by intravenous thrombolysis were significant (Table 3). Finally, the logistic model was statistically significant (Table 4). The RDW values were independently associated with a higher risk of developing symptomatic hemorrhagic transformation with an adjusted OR of 1.9(95 % CI 1.37-2.93, P < 0.001). In addition, the NHISS score on admission (adjusted OR 1.1, 95 % CI: 1.04-1.16; P = 0.001) was a significant predictor for sHT outcome.

**Table 3:** Univariable logistic regression analyses showing the associations of RDW level on admission and other baseline characteristics with HT; sHT.

**Variables**	**Hemorrhagic transformation**				**Symptomatic hemorrhagic transformation**		
	**OR**	**95%CI**	**P-value**		**OR**	**95%CI**	**P-value**
Age	1.048	1.011-1.087	0.011		1.03	0.98-1.08	0.286
Men(n, %)	0.82	0.405-1.658	0.58		1.37	0.46-4.1	0.578
Hypertension(n,%)	0.793	0.356-1.768	0.57		2.77	0.61-12.56	0.186
Diabetes mellitus(n, %)	0.733	0.333-1.613	0.441		0.84	0.23-3.08	0.8
Hyperlipidemia(n, %)	2.314	1.045-5.125	0.039		1.427	0.475-4.29	0.527
Consumption of alcohol	0.569	0.227-1.43	0.23		1.55	0.51-4.7	0.44
Smokers/ex-smokers(n,)	0.603	0.272-1.339	0.214		0.69	0.21-2.21	0.53
Previous stroke or TIA (n, %)	0.9	0.329-2.46	0.837		1.44	0.39-5.35	0.58
Atrial fibrillation(n, %)	2.749	1.31-5.769	0.008		1.92	0.63-5.84	0.25
Coronary artery disease(n, %)	0.507	0.191-1.344	0.172		1.61	0.2-12.74	0.65
Onset-thrombolysis time(minutes)	1.002	0.995-1.009	0.537		1.008	0.99-1.02	0.14
NIHSS on admission	1.104	1.058-1.152	< 0.001		1.108	1.05-1.17	< 0.001
RDW	2.863	1.935-4.236	< 0.001		2.002	1.37-2.93	< 0.001
Platelet counts	0.992	0.985-1	0.038		2	1.37-2.92	< 0.001
BG on admission(mmol/L)	1.004	0.873-1.155	0.956		0.98	0.78-1.22	0.83
SBP on admission (mm/Hg)	0.982	0.966-0.999	0.034		0.99	0.97-1.02	0.52
DBP on admission(mm/Hg)	0.998	0.972-1.024	0.865		1.01	0.97-1.05	0.53

TIA, Transient ischemic attack; NHISS, National Institutes of Health Stroke Scale; HT, hemorrhagic transformation; BG, blood glucose; SBP, systolic blood pressure; DBP, diastolic blood pressure; RDW, Red blood cell distribution width.

**Table 4:** Multivariable logistic regression analyses showing the associations of RDW level on admission with HT; sHT.

**Variables**	**HT**			**Symptomatic HT**	
	**Adjusted OR(95%CI)**	**P-value**		**Adjusted OR(95%CI)**	**P-value**
RDW	2.5(1.73-3.71)	<0.001		1.9(1.37-2.93)	<0.001
NIHSS on admission	1.08(1.03-1.14)	0.002		1.1(1.04-1.16)	0.001

NHISS, National Institutes of Health Stroke Scale; HT, hemorrhagic transformation; RDW, Red blood cell distribution width.

**Figure 2:**
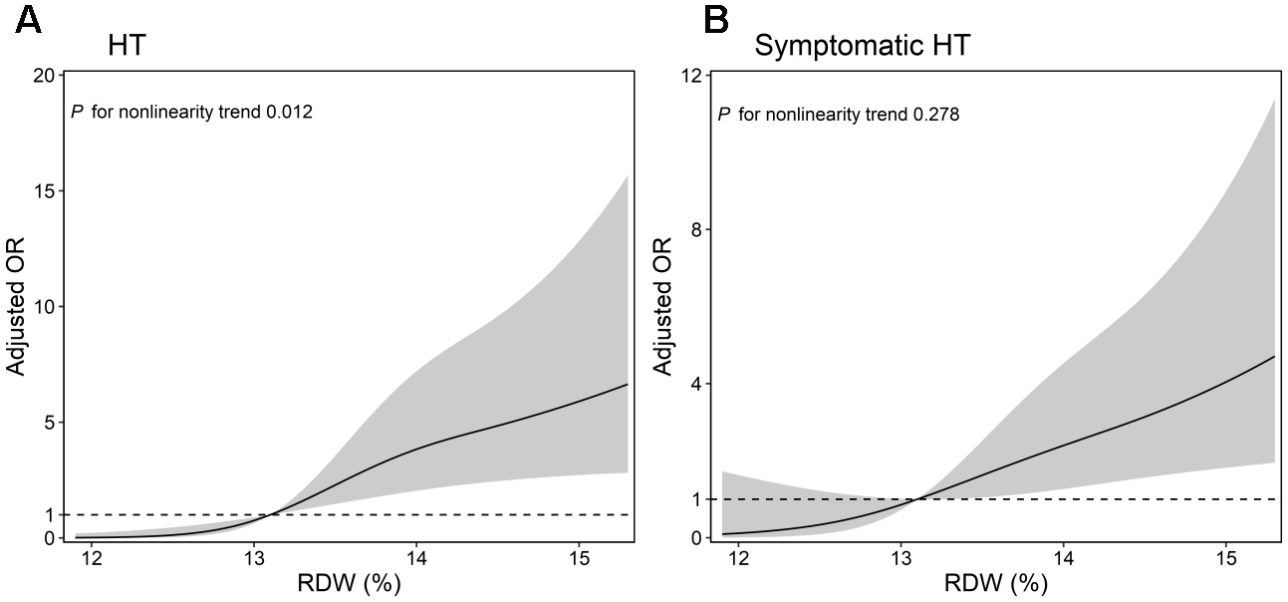
Multiple-adjusted restricted cubic spline regressions were used to analyze the association between RDW and risk of HT, (**A**) symptomatic HT (**B**) after IVT placed at three knots (at the 10th, 50th, 90th percentiles). The solid line represents adjusted odds ratios, while the shaded area represents 95% confidence intervals (CI). Reference point for RDW was the median (13%). RDW, Red cell distribution width; HT, hemorrhagic transformation.

Multiple-adjusted restricted cubic spline regression results showed that the pattern and magnitude between elevated RDW levels and the risk of HT and symptomatic HT (Figure 2). The adjusted OR of HT and symptomatic HT increased in a dose-dependent manner according to increasing levels of RDW. The odds ratios for HT were adjusted for age, atrial fibrillation, hyperlipidemia, NIHSS, platelet count and RDW, while those for symptomatic HT were adjusted for age NIHSS and RDW.

**Figure 3:**
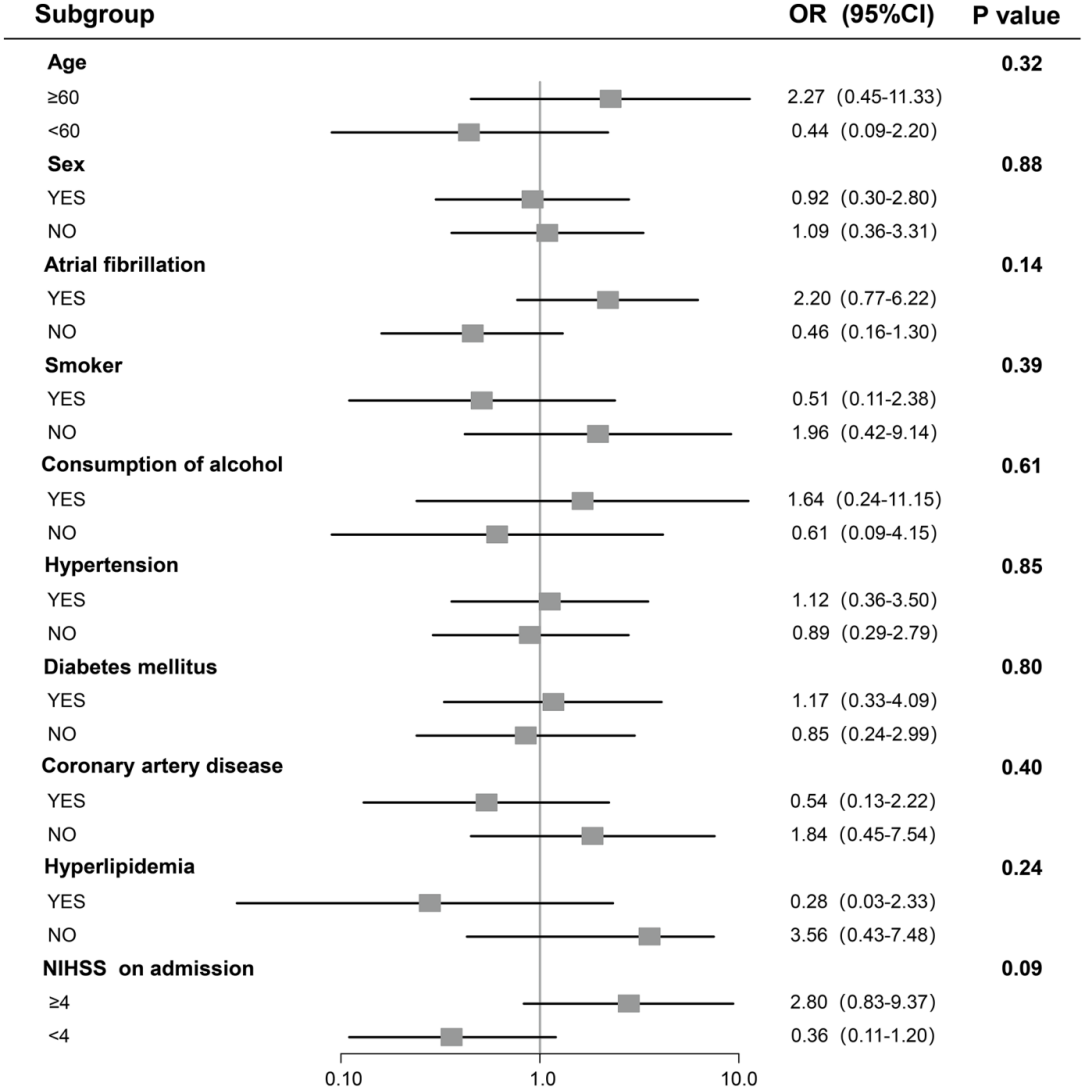
**A Result of stratified logistic regression analysis testing association between RDW and hemorrhagic transformation.** For subcategories, black squares represent OR, and horizontal lines indicate 95% CI. For baseline NIHSS score, subgroups were dichotomized by median value.

Stratified logistic regression analysis (Figure 3) used to identify variables, age (<60 versus ≥60), sex (male versus female), atrial fibrillation, smoker, hypertension, consumption of alcohol, diabetes mellitus, hyperlipidemia, coronary artery disease and baseline NIHSS score (< 4 versus ≥ 4), that modify the correlation between RDW levels and HT. Since all the P values for interaction were >0.05 and all the confidence intervals crossed the OR=1 reference line, all tests for interactions were not significant.

## DISCUSSION

To the best of our knowledge, this study provides the first comprehensive assessment of the relationship between RDW and HT or sHT after IVT in real-life situations. In this single-center retrospective study of AIS patients treated with IVT, we found that the incidence of HT and sHT was 12.6% and 5.2%, respectively. In our study, RDW was significantly associated with hemorrhagic transformation and symptomatic hemorrhagic transformation in patients who underwent intravenous thrombolysis for acute ischemic stroke in models adjusted for age, AF, platelet count and NHISS on admission.

RDW is a simple, rapid, and widely available method for assessing RBC size heterogeneity, and it is calculated using the standard deviation (SD) of RBC volume. The known minimum and maximum values of RDW are 11% and 15%, respectively [15]. Clinically, pathological increase in RDW is associated with anemia caused by iron, folic acid or vitamin B12 deficiency [16]. RDW values are also increased in some autoimmune diseases, myelodysplastic syndrome, hemolytic anemia, liver dysfunction, sickle cell disease, and blood transfusion therapy [17]. Recently, several studies have shown that increased RDW levels are associated with an increased risk of coronary heart disease, atrial fibrillation, carotid artery atherosclerosis, and ischemic stroke [18–22].

Several studies have focused on the association between RDW levels and functional outcomes after ischemic stroke. In previous studies based on stroke population, high RDW levels are an independent prognostic marker for 3-month functional outcome [23] and 30-day mortality [13]. A recent study found that RDW levels are significantly correlated with the severity of neurological function, which may be an important prognostic marker for AIS patients [24]. We also observed a significant association between RDW levels and 3-month functional outcomes. In our study, the patients with high RDW levels had lower rates of favorable functional outcome compared to patients with lower RDW levels in both genders (all p<0.05).

In a recently published study, the risk of HT increased stepwise across RDW tertiles. Reperfusion therapy can modify the relationship between RDW and HT, with no significant association being observed between RDW and HT among patients who underwent reperfusion therapy [14]. However, reperfusion therapy includes IVT and EVT, yet the study did not specifically assess the relationship between RDW and HT in patients who underwent IVT. Therefore, EVT could have been a confounding factor in the study. As a result, the relationship between RDW and HT in patients that undergo IVT is unknown. Our study provides detailed information about hemorrhagic transformation after thrombolysis, an aspect that has not been investigated in previous studies to the best of our knowledge. We also found that the relationship between RDW and HT after IVT was independent of other known predictors of hemorrhagic transformation such as infarct volume, and NHISS on admission [3].

The rt-PA reperfusion process causes oxidative stress and release of proinflammatory cytokines leading to the blood–brain barrier (BBB) damage. Furthermore, the process of rt-PA itself can aggravate BBB disruption and increase the risk of HT [25]. A study carried out on animals showed that neuroinflammation results in ischemia-induced HT by inducing metalloproteinase -9 activation [26]. The study showed that oxidative stress mediates BBB damage via MMP activation in mice with copper/zinc-superoxide dismutase deficiency [27] and that treatment with the free radical scavenger significantly reduces rt-PA induced HT in embolic focal ischemia [28]. This indicates that oxidative stress plays an important role in BBB dysregulation during HT in the ischemic brain. Anoxia is also known to increase BBB permeability in cerebral capillaries [29].

The exact pathophysiological mechanism of the relationship between RDW and developing hemorrhagic transformation in patients who have undergone IVT is unknown. RDW levels have been reported to be associated with oxidative stress after 2h of focal cerebral ischemia, with an increase in reactive oxygen species levels in microvessels and astrocytic endfeet [30]. Oxidative stress could increase RDW by inhibiting red blood cells production, increasing the size imbalance of red blood cells, and changing the deformability of cell membranes [31]. Elevated RDW levels are correlated with low oxygen exercise capacity [32], which could exacerbate HT development. Red blood cell distribution width is also correlated with hsCRP [33], and could be a potential biomarker of inflammation in stroke patients. AIS increases the secretion of inflammatory cytokines, inhibits genes expression involved in iron metabolism and hemoglobin synthesis and induces an increase in RDW levels [34]. An increase in RDW levels causes acute hypoxia, inducing EPO-driven erythropoiesis and a rapid increase in erythrocyte size variability [35]. Inadequate oxygen supply causes an increase in the blood-brain barrier permeability, leading to uncontrolled vasogenic edema, microvascular ischemia, or hemorrhagic transformation [36]. Therefore, RDW may be a marker for detecting hypoxia and oxidative stress. Future studies should systematically explore the mechanisms underlying the effects of RDW levels on HT.

A recent study found that there is a positive correlation between RDW and serum neuron-specific enolase levels. There is also an independent positive relationship between RDW and neuronal damage in AIS patients after adjusting for potential confounders [37]. A study by Lan Hong et al. demonstrated that increased baseline RDW is associated with poor collateral flow and increased final infarct volume in patients with large artery atherosclerosis stroke [38]. Since poor collateral flow and infarct volume have been known to cause hemorrhagic transformation [39], we believe that many factors could contributor to poor prognosis in AIS patients. These factors include some blood biomarkers associated with inflammation and oxidative stress that are readily available and can be included in the clinical scoring systems to predict clinical outcomes. Besides, many factors are changeable and can be targeted for therapeutic intervention. Based on our study results, we propose the use of RDW as a laboratory parameter for automatic routine hemogram detection. RDW can be used to predict hemorrhagic transformation in AIS patients with intravenous thrombolysis. Early triage, diagnosis, and management are compelling needs for the management and care of patients with cerebral ischemia.

Our study has some limitations. First, this is a single-center based retrospective analysis, with the limited data influencing the statistical significance of our conclusions. Therefore, further research in larger samples is needed to minimize the selection bias. Second, the numbers of HT and sHT cases were too small to perform an HT subtypes analysis further. Third, we lacked dynamic RDW data, that could reflect the dynamic nature of the inflammatory state. These could be the focus of our future studies. The current analysis mainly explored the association between RDW and HT in IVT patients, and to assess the risk of HT due to RDW requires further work. Nevertheless, the strength of our study reported on baseline RDW levels in acute ischemic stroke patients treated with intravenous thrombolysis.

In summary, we found that high red blood cell distribution width levels could increase the risk of hemorrhagic transformation after IVT. We think that the association between RDW and hemorrhagic transformation in AIS patients treated with intravenous thrombolysis, could be due to the mechanism of reactive inflammation and oxidative stress state.

## MATERIALS AND METHODS

Acute ischemic stroke patients who received rt-PA thrombolysis in Northern Jiangsu People's Hospital between 1 January 2017 and 31 December 2019 were consecutively recruited into this study. The inclusion criteria were as follows: (1) adults (age, >18 years), (2) The diagnosis of AIS based on World Health Organization criteria, and confirmed using magnetic resonance imaging (MRI), and (3) occurrence of stroke symptoms within 4.5 hours and the use of rt-PA therapy. (4) informed consent from patients or their relatives.

Exclusion criteria were listed as following: (1) history of craniocerebral operation, (2)contraindications of thrombolytic therapy including treatment with low-molecular-weight heparin within 24h, active hemorrhage, aortic dissection, platelet count of < 100 x 109/L in the peripheral blood; (3) anemia caused by previous diseases of the blood system and other causes; (4) autoimmune diseases, malignant tumors, congenital heart disease, hyperthyroidism, severe liver and kidney dysfunction; (5) a clear history of infection before admission; (6) history of blood transfusion and use of iron, folic acid and vitamin B12 within three months; (7) patients without sufficient data from laboratory examinations, or follow up information. Ethical approval for this study was obtained from the Ethics Committee of Northern Jiangsu People's Hospital. According to the declaration of Helsinki, written informed consent was obtained from each study participant.

### Treatment administration

Intravenous rt-PA was used at a dose of 0.9 mg per kilogram (maximum of 90 mg), with 10% of the total dose being used as an initial bolus within 1 minute and the remaining dose administered as a constant infusion for 60 minutes.

### Data collection

Imaging findings, blood test results on admission, the National Institutes of Health Stroke Scale (NIHSS), (which was used to assess the severity of stroke on admission), onset-to-treatment time (OTT), demographic data and medical history including age, sex, and history of hypertension, diabetes mellitus, hyperlipidemia, smoking, atrial fibrillation, and previous stroke/TIA were collected. The initial RDW levels determined on admission before thrombolysis were also collected. Two researchers blinded to this study independently analyzed all the data, and a third researcher resolved any disagreement between the two researchers. Ethylenediaminetetraacetic acid (EDTAK2) anticoagulation vacuum tubes were used to collect 2ml of venous blood. The XE-5000 automatic blood analyzer of Sysmex Company was used for RDW analysis.

Head CT scans were repeated 24 h after treatment with IVT and another CT scan done immediately in case of clinical neurological deterioration to assess the existence of hemorrhagic transformation. Two neurologists experienced in neuroimaging who had not seen the patients' clinical data, independently evaluated the imaging findings.

### Classification criteria

The definition of hemorrhagic transformation is that no hemorrhage was observed in the first cranial CT/MRI after cerebral infarction, but intracranial hemorrhage was observed on the second cranial CT/MRI examination [9]. CT scans were used to confirm and categorize hemorrhagic transformation. Symptomatic hemorrhagic transformation (sHT) was defined as an increase of the total NIHSS scores by more than 4 points compared with the score on admission according to the Heidelberg Bleeding Classification [40]. Smokers were defined as individuals who smoked at least one cigarette per day for at least a year and were current smokers. Alcohol consumption was defined as consuming 1 or more alcoholic drinks per day during the previous year. Functional independence was defined as a score of 0-2 points on the modified Rankin Scale, at three months of follow-up.

### Statistical analysis

Continuous variables with normal distribution were expressed as mean ± standard deviation, while variables that did not conform to normal distribution were represented by using median and interquartile range (IQR). Patients were divided into two groups based on gender since RDW levels had been reported to be higher in females than in males [41]. Student’s t-test was used to analyze data that showed normal distribution, otherwise the data was analyzed using the Mann-Whitney U-test or Kruskal-Wallis H-test. On the other hand, the difference between categorical variables was analyzed using the Chi-square test or the Fishers accurate test. Variables with P < 0.10 in univariate analysis were candidates for stepwise forward multivariable logistic regression analysis to identify the independent predictors of outcome. In addition, we used restricted cubic splines with three knots placed at the 10th, 50th, and 90th percentiles to evaluate the pattern and magnitude of RDW with hemorrhagic transformation and symptomatic hemorrhagic transformation. Stratified logistic regression models were used to perform subgroup analyses. All statistical analyses were performed using Stata 15.1 (StataCorp LP, College Station, TX, USA), and R version 3.6.3 (R Foundation for Statistical Computing, Vienna, Austria). A two-tailed p-value of less than 0.05 was considered to be statistically significant.
